# Blind search and flexible product visions: the sociotechnical shaping of generative music engines

**DOI:** 10.1007/s00146-024-01862-x

**Published:** 2024-03-01

**Authors:** Oliver Bown

**Affiliations:** Interactive Media Lab, School of Art and Design, UNSW Sydney, Sydney, Australia

**Keywords:** Creative AI, AI music, AI start-ups, Generative music engines

## Abstract

Amidst the surge in AI-oriented commercial ventures, music is a site of intensive efforts to innovate. A number of companies are seeking to apply AI to music production and consumption, and amongst them several are seeking to reinvent the music listening experience as adaptive, interactive, functional and infinitely generative. These are bold objectives, having no clear roadmap for what designs, technologies and use cases, if any, will be successful. Thus each company relies on speculative product visions. Through four case studies of such companies, I consider how product visions must carefully provide a clear plan for developers and investors, whilst also remaining open to agile user-centred product development strategies, which I discuss in terms of the ‘blind search’ nature of innovation. I suggest that innovation in this area needs to be understood in terms of technological emergence, which is neither technologically determinist nor driven entirely by the visions of founders, but through a complex of interacting forces. I also consider, through these cases, how, through the accumulation of residual value, all such start-up work risks being exapted for more familiar extractive capitalist agendas under the general process that Doctorow calls “enshittification”. Lastly, I consider a number of other more specific ways in which these projects, if their growth is achieved, could influence music culture more broadly.

## Introduction

In this paper, I consider how a number of music AI start-ups are attempting to undertake a long-prophesised revolution in music technology in which the “fixed media” form of recorded music is replaced by dynamic and adaptive streams of *generative* and *interactive* music, and examine what the potential impacts of this commercial drive are for music culture. I do this through a comparative study of four organisations—three start-ups and one music production studio—each developing their own in-house software frameworks for the dynamic mixing and streaming of music. I loosely categorise these frameworks as “generative music engines”, abbreviated as GMEs.

These companies are part of a surge of interest in commercial applications of AI to music, fuelled by a bullish investment interest in new AI applications. There is reasonable expectation that such commercial GME creators could become major music platforms. Even if they fail at this, such start-ups have the potential to develop other valuable technologies and assets that could make them worthy of acquisition. Venture capital has been keen to secure a foot in the door in any such market breakthrough. In 2019, for example, high-profile tech investor Vinod Khosla, claimed that algorithmically generated music that responds to our mood will rapidly and completely displace recorded music.^1^

Yet, GMEs are a highly speculative area for commercial success. Unlike many application areas for AI, where there is a clear user-need or “pain point” that new technology would solve, there is no obvious market need that is fulfilled by GMEs. They are significantly more complex than existing media production and distribution methods, presenting an array of complex interconnected, design and creative-practice problems to solve, and they also face a “cold-start” problem: in order to be profitable, they must build a community of users in a previously unexplored area, at the same time as developing new software designs and accumulating content, resulting in “product-market fit”.

This potentially radical impact on music culture from the commercial sector, speculative though it is, warrants a better understanding of how start-ups are tackling this complex innovation landscape. This is particularly important because commercial products, platforms and cultures of practice can become rapidly locked in once they have become established, and because large commercial platforms have widely discussed downsides including hidden data extraction practices, effective monopolies (Crawford 2021), and a tendency towards what Cory Doctorow calls “enshittification” (Doctorow 2023): the doubling down on profits through reduced service quality after an initial honeymoon period.

In this paper, I investigate the work of four companies developing GMEs, looking at the products they are developing, how they communicate a value proposition about generative music, and how they approach innovation, especially the need to be open to unexpected innovation opportunities. The result is a detailed snapshot of the present commercial search for successful GMEs. I then analyse this snapshot from a sociotechnical systems perspective in which the form of GMEs and related products are understood as emerging through a complex interaction of factors and competing actors. This analysis is used to critically examine how commercially driven start-up culture potentially impacts creative music practices more broadly. In this I align with Georgina Born, who in a recent paper (Born 2022a, b) argues that multiple relationships between music, AI and culture are simultaneously at play. Specifically, Born both welcomes and points to the limits of a Foucauldian perspective (and specific recent work such as Prey 2018; Drott 2018; Stark 2018)) that prioritises attending to the commercial world’s power over its consumers, and the need to maintain critical oversight over such power. She balances such a perspective with the need to understand culture as shaped through more chaotic and less controlled interacting forces, resulting in emergent phenomena, where commercial initiatives are constrained by the unpredictable nature of technologies and users.

This paper attempts to capture and develop an understanding of this dynamic, in which founders balance the pitching of bold product visions with the need to be flexible and responsive to their unfolding understanding of technologies and users, and the ever-present option of being acquired. It critically examines what potential impacts this dynamic of commercial innovation has on the unfolding of new cultures of music production and consumption. It considers how the visions presented by founders, that emphasise a benefit to society by enhancing and enriching music culture, play out under the commercial pressures placed on start-ups under the conditions of their funding.

Methodologically, I construct this snapshot through interviews with founders and developers, and archival and online research of company websites, profiles, public media articles and user forums. Additionally, in the case of Uncanny Valley, with whom I collaborate, I include participant observation. Due to limited access to technical details of each company’s technology, my understanding of their GME designs is partly speculative, but I bring my own years of experience working with GME architectures and constraints to make reasonable guesses about these system implementations.

These case studies serve two core questions, both of which centre around how companies are presenting value propositions and product visions: Q1 how do start-ups manage innovation in such a speculative field? Q2 how does their own conception of value play out in the decisions they make? I will elaborate on these questions below through two thematic discussions. More broadly, these two questions relate to issues of autonomy, and what forces are truly responsible for shaping sociotechnical outcomes. This informs a discussion about the potential risks and opportunities for music culture.

This paper proceeds by introducing GMEs (Sect. 2), then discussing the two themes related to questions (1 and 2) (Sects. 3 and 4). Then I will present the four case studies (Sect. 5), followed by some pairwise comparisons between the organisations (Sect. 6). The questions are discussed in Sect. 7.

## What is a generative music engine?

The use of algorithms to generate music has a long history in academia and experimental arts (Dean 2018), but until recently limited commercial application. As algorithmic content generation technologies have advanced, new commercial applications have come into view. The focus of this paper is the specific subdomain of commercial music technology companies which seek to create new forms of generative, reactive and interactive music experiences. This author could identify well over 100 companies working at the AI music intersection, including several that have received many tens of millions of dollars in funding. Several of these are working in new forms of music distribution and consumption. Amongst those, the companies that are considered here are prominent. Conceivably, by innovating successful new music consumption experiences, an emerging company such as these could be the next Spotify, with immense potential for holding attention, stimulating participation, performing personal data extraction, and controlling music markets.

Influential visions of the impact of generative music on music experience have shaped this debate for some time. In the 1990s, Brian Eno asked if the production of static musical recordings might soon be displaced by generative music as the dominant form of music listening experience (Eno 1996). “You mean you used to listen to exactly the same thing over and over again?” he imagines his grandchildren asking him. More boldly, Ray Kurzweil predicted in 1999 that by 2019 AI artists would stand as equals alongside human artists (Kurzweil 1999). These longstanding commentaries indicate that the question of forms of generative music experience have been in mind and conceptually well explored. But only relatively recently has there been any significant push to develop products that achieve this reality, including commercial, open source and academic initiatives.

In the past few years, there has been a rapid shift powered in part by advances in AI, but arguably more so by the evolution of the data infrastructures and cloud and mobile platform ecosystems that make new forms of generative and networked musical experience practicable (Crawford 2021). i.e., now that everybody has a powerful cloud-networked computer with realtime audio capabilities on their person, innovative AI music experiences have a market in waiting. (As discussed below, (Drott 2021) also proposes a basis for this expansion in the circumstances of global capital). The companies covered in this paper (whose applications are discussed below) each have in common that they are working to create dynamic music experiences in which original musical passages are mixed and modified either via generative processes or interactively by users. These companies are working to build large datasets of musical elements, along with a structured engine that arranges and sequences this music according to musical rules and input parameters.

A GME, then, is a system consisting of realtime rendering software, musical assets, other backend software, and associated organisational strategies and workflows, that can be used to deliver final music tracks dynamically. This definition includes very simple systems that don’t overtly use AI. In the simplest case, a dataset of audio loops of drum-beats, basslines, chord lines and melody lines could be played back through a four-track looper picking parts according to simple algorithmic rules. Such systems have existed in the gaming world for years, used to make reactive soundtracks for games.

**Fig. 1:**
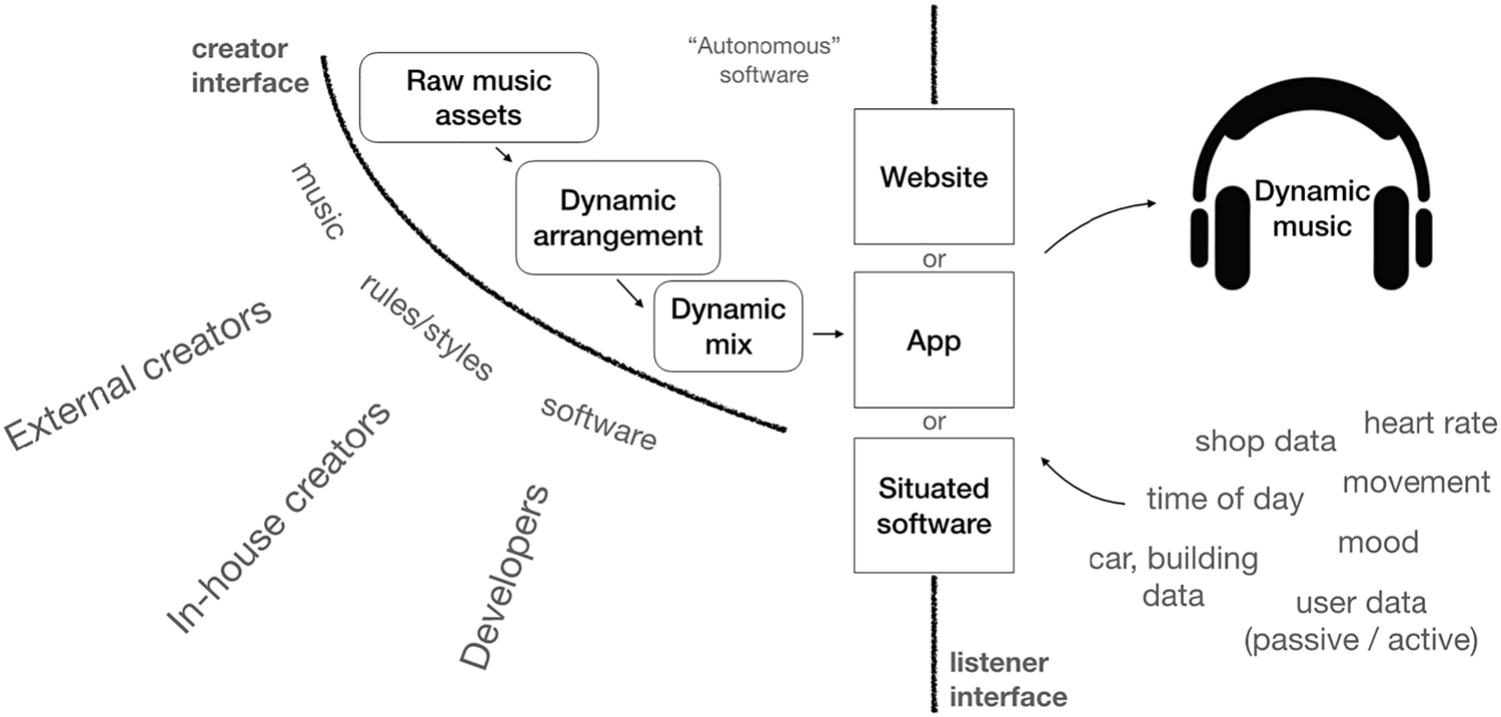
Generic depiction of the sociotechnical architecture of a generative music engine (GME). See main text for details

Specifically (Fig. 1) , a GME supports the dynamic generation of musical content, personalised for a listener, and potentially utilising a range of real-time data sources such as movement, time of day, physiological data or data from the built environment (right). It is delivered through real-time software (the GME itself) that composes, arranges and mixes music either on the cloud (server side) or on a device (client side), such as through a website, app or situated computer program (such as in a hotel lobby) (Fig. 1, middle vertical line). GMEs utilise a combination of raw music assets, which may be in audio, symbolic, rule-based or other algorithmic forms, and software elements that perform dynamic arrangement and mixing (Fig. 1, top left). Musical elements may be created by 3rd party external or in-house creators, and software elements may be created by 3rd party or in-house developers (Fig. 1, bottom left). Raw music assets may also be compiled or created using machine analysis or generation processes, such as analysing existing music to create a database of chord progressions.

Some generative music systems may make heavy use of AI, but we can consider the AI and the engine itself to be orthogonal elements. GMEs are broad frameworks in which AI may be used in a range of ways, from generating melodies, to running offline batch-processing of audio files for tagging purposes. Indeed, they are very much in flux in relation to the promise and use of AI; a GME may be general enough to have the capacity to switch between a stable dataset of pre-generated melodic lines, and a more experimental, risky melody generator which is being incrementally improved. In the world of “software-as-a-service”, companies are adept at building highly modular programs with many swappable elements.

Relatedly, a broad observation with these and other companies is that when they cite the power of AI in their marketing, it is typically hard to pinpoint what it is being used for.^2^ AI is seen as supporting the credibility of effective dynamic music generation, but such a perception can be expected to evolve as the technology and our relationship to it changes. This will be explored in the following examples.

## Start-up innovation in GMEs

Innovation is inherently speculative and exploratory, but as the above section makes clear, GMEs represent a strong example of a complex design space in which multiple factors—economic, cultural, technical—must be simultaneously addressed, with is no clear and fixed measure of what makes a particular GME design good. Technically, GMEs are deceptively simple in one sense: often the user experience is as simple as clicking on a stream and listening to it. But under the hood they present myriad technical design decisions. Considering the realtime rendering engine alone in terms of just one of many examples, decisions need to be made about whether digital audio effects (like reverb and EQ) will be applied to individual tracks in realtime, bearing in mind that rendering may need to happen on limited resources such as smartphone CPUs. Any such decisions may have radical impacts down the line: a limited suite of effects may put off artists from using the tool; but including effects may present problems for how well music plays on smartphones.

How exactly a GME mixes musical elements is a central issue, since this is the primary aspect being taken out of the hands of music producers and automated: should it implement a single algorithm applied to all musical styles?; should individual artists be able to specify rules about how their music is mixed?; could AI be used to listen to, evaluate and improve the resulting mixes? Whether a GME is intended to work with material composed by multiple human artists, or fully AI-generated material, these are problems that remain to be solved, and for any given solution, further usability and interaction design questions arise. At the other end of the pipeline lie sociocultural and aesthetic issues: who will listen to GME-created music? Where, when and why? What are the criteria for a successful experience? How will it be paid for? How will it compete with existing music streaming experiences? Back again at the creation end: will artists embrace music creation for GMEs? Do GME creation tools need to be integrated into existing digital audio workstations (DAWs)?

Innovation in this area is thus both routine and extraordinary; it is one thing to envision a new AI-powered music product, but another thing altogether to make it work, and create a market for it, where no user need is immediately evident.

How, then, do GME developers, and the wider innovation ecosystem they inhabit, manage technical innovation, social discourses about their value and product visions, and other competing constraints?

This can be framed by two themes relating to risk in start-up culture. Firstly, fundamental to start-up culture is the idea that individual start-ups have a low chance of success, but a potentially very high yield if successful. Investors, particularly venture capitalists (VCs), are prepared for the fact that most companies they back will not survive, while a handful may produce 10 ×, 100 ×, 1000 × or more returns, outweighing the losses. The other theme is that the start-ups themselves, if run well, will constantly monitor and adapt to what they learn about user needs in search of a “product-market-fit” (Seibel 2018). A complex of design and innovation methods, including agile software development, human-centered design, and rapid prototyping, has arisen in start-up innovation communities to address this challenge. A key principle here is to draw on the humility of the scientist, that only by listening and responding to evidence can great products be created. Yet, innovation leaders must lead with strongly directed product ideas.

**Fig. 2:**
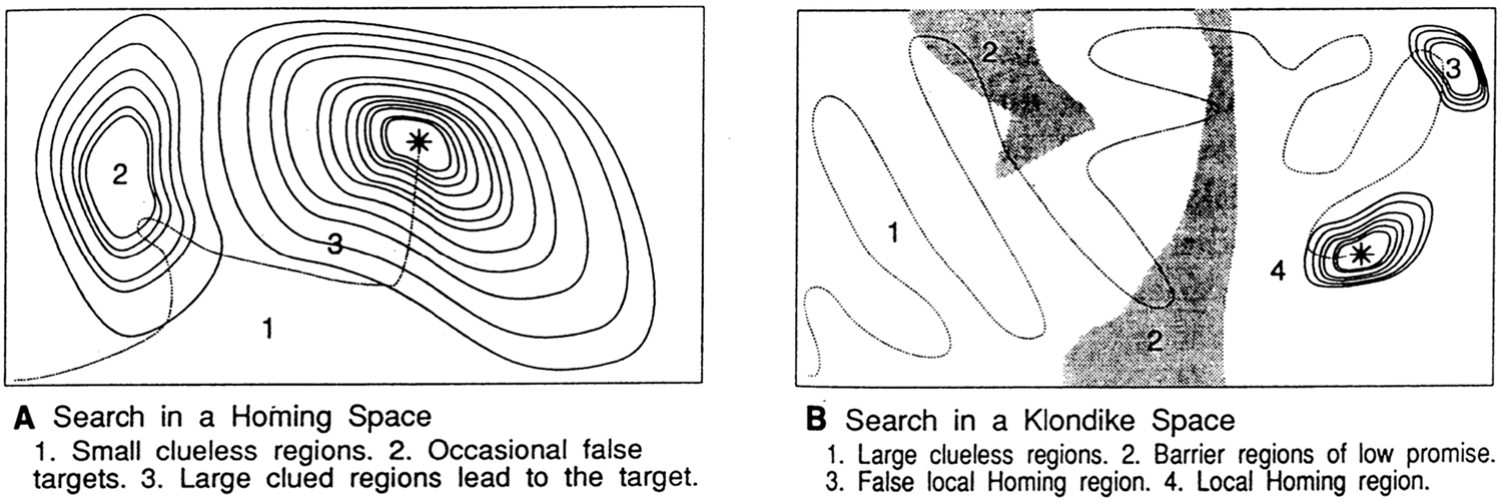
From Weber and Perkins (1992). Homing using local information can happen in some creative contexts (**A**) but more generally it is blind (**B**)

Both themes channel the fundamentally “blind search” nature of creativity and innovation, at the heart of which is the idea that in previously unexplored domains innovators cannot possibly know which path will take them to a winning solution (Perkins 1996; Simonton 2011; Stanley and Lehman 2015). Accordingly, the most powerful strategy in innovation is not to plan a path to a successful design, but to allow informed but partially undirected search to take place. The concept is compellingly expressed through a spatial metaphor^3^ by Weber, Perkins, et al. (Weber and Perkins 1992), where a goal is represented as a point in a geographic domain, the location of which is not known, like hidden treasure (Fig. 2). They compare “homing spaces”, where local information points to the solution, with “Klondike spaces”, named after the Klondike gold fields, where local information may give no clues, or even be misleading, as to where a productive solution lies. Such logic leads to well-known heuristic search strategies outlined in several key texts on creativity (Boden 1990; Sternberg and Lubart 1991; Amabile 1996; Sternberg 2006; Simonton 2011), and echoed in start-up doctrine.

Accordingly, the VC invests in founders, their teams and their visions, but they spread their bets. Specific successes and failures have low predictability; the investor thinks of the statistics of success and failure across a larger number of companies. An investor sees that AI will surely transform music delivery, but can’t imagine what form that disruption will take, so they scan competing alternative visions (YCombinator 2019). Likewise, individual companies must maintain one or more product visions which direct their development, but by working in an agile, iterative manner with user needs as their focus, can keep scanning for productive changes to that vision. The epitome of this adaptive, search-based thinking can be found in the principles of the “Lean Start-Up” model (Eisenmann et al. 2012).

A potential point of friction, then, lies in the need for founders to commit to clear product visions—as presented to investors, employees and customers—whilst remaining agile. Given the potential value of blind, exploratory search, how should we understand the untested visions of companies for their generative music products? Are they literal visions of future products, or can they be treated as looser placeholders that establish the conditions for more blind, experimental forays into the unexplored spaces of music technology? A fruitful way forward is that they should be seen as both, and that any vision can be read both in terms of its literal playing out, and in terms of its potential to create the conditions for further agile innovation.

As illustrated in the following sections, the AI music domain has an especially large number of start-ups that have very speculative value propositions, perhaps supported more by conviction than by evidence of a user-need. This keenness to put exploration first was anecdotally reinforced by a friend recently embarking on a music AI start-up who stated this explicitly: their plan was start by building a generative system and then explore its potential uses, of which there was a wide range of possibilities (personalisation, focus music, branding and so on). *Once they had the basic engine set up*, they would be in a position to explore all these use cases through rapid prototypes and user-centred design. In the absence of a clear goal, or developed user testing framework, the initial engine design could only come down to the developer’s intuition, immediately shaping the development journey in undirected ways.

Thus, both within and between individual start-ups, innovation can be understood as a tussle between a more concrete product vision and a placeholder vision which allows for aspects of blind search to take place. Here, I take inspiration from Hodgson’s (Hodgson 2020) work on the concept of imagined metrics, also applied to music technology start-up companies. Hodgson shows how the metrics describing a company’s predicted performance are frequently knowingly exaggerated by CEOs, investors and staff, an open secret that is nevertheless useful for rallying commitment, investment, and enthusiasm. As Hodgson details, this is more than a simplistic or cynical drive to over-value companies, but more functionally stands to steer the company towards profitable opportunities. In this way, imagined metrics themselves might even be seen as a critical mechanism for forcing more open-ended search ambition in start-ups.

This theme gives context to this paper’s first research question, now stated as follows: how is the power of blind search manifest in the companies’ strategies, and how do they reconcile agility with a clear direction?

## Acquisitions, pivots and extraction

Bound up with the question of how innovation is treated is the multifaceted nature of a company’s value. The primary focus on a company’s value is the value of the product it is busy creating, but value also exists in the accumulation of a user base, and the construction of teams, culture, user-centred knowledge, and diverse assets ranging from tables and chairs to collections of tagged audio samples, patents, user data and lines of code. A company can fail to produce the product it set out to make but still have significant accumulated value, potentially being successfully acquired for reasons not directly related to its primary product offering.

Amongst this array of sources of value, the value of people, and their organisation into teams, is particularly important, as voiced by investors and managers. VCs stress the character of their founders, even suggesting that the particular details and plausibility of a pitch are less important than the capability of the founder to inspire a vision (Cannon 2019; Drott 2021). Similarly, CEOs and other leaders are strongly focused on building the right teams, working together in the right way. Even if a product is not a commercial success, the team may still have proven value.

Acquisition is a natural outcome for start-ups, and notable acquisitions have already occurred in the music AI space. UK AI music start-up Jukedeck was acquired by Bytedance in 2019 and another start-up from Abbey Road Red in London, called, confusingly AI Music, was acquired by Apple in 2022. In both cases, the company then ceased to exist and the product vision the company was offering disappeared into the acquiring company. The respective acquiring companies redeployed the teams, IP, databases and so on. User agreements with standing users may have been updated in the process (Drott 2021). Such moments potentially have great significance: privately owned software developed according to a particular vision and set of values can easily detached from that vision and set of values.

In comparison, the musical instrument company Roli, with several performing products, overcapitalised and filed for bankruptcy in 2021 (Seah 2021). They had overestimated the market for instrument concepts that turned out to be more specialised than they anticipated. In response, they pivoted their business to music education products (combining media and interfaces), where a wider mass market could be identified.

Thus, diverse forms of value are a daily factor in decision making. Accumulating a database of audio samples, metadata and software infrastructure may be a distraction from *real* innovation but if well managed, is a value-accumulating activity. Added to this, putting together innovative teams of musicians and software developers, a new collective entity empowered to break ground in music technology applications, is arguably the most important innovation work these companies can do.

This relates to start-up founders’ visions and values. Each operates knowing that if their primary product visions fail, or even if they succeed, an outcome may involve acquisition by companies who do not share the same values, and particularly those whose core business is data extraction. Thus, whilst, in terms of problem solving and search, start-ups can be understood as an effective way to divide up the work of solving needs in society (how start-up infrastructures are commonly promoted and justified to governments), they are positioned in ecosystems where noble aims can be easily converted to extractive goals (Drott 2021; Watson and Leyshon 2022).

Drott highlights this generative force in recent work on AI music start-ups, by considering how extractive capitalism seeks innovative markets:

“[a] key factor driving the growth of commercial AI has been the considerable financial investment that has been staked on such ventures. This is of a piece with the massive injection of capital into AI-related research and development that has taken place since 2010, itself tied to broader political-economic tendencies: a growing mass of surplus capital in search of profitable sites of investment; the long-term decline in productivity in capitalist economies worldwide; and, concomitantly, the search for technical or other fixes that might reverse these trends and relaunch a new wave of capital accumulation. ... This influx of capital into AI in general and music AI specifically represents a wager, one staked on its future profitability. Whether or not this wager pays off, it is already having an impact, by reshaping perceptions of what music AI is or should be.” (Drott 2021).

Thus, we might analyse the problem of understanding start-up innovation and impact in music as one in which product visions are not only ambiguous, but are vulnerable to a sort of systematic exaptation. Within this, the founders’ and company employees’ immediate vision and innovation work may be subverted by competing forces.

This theme gives the context for the paper’s second question: how do diverse conceptions of company value play out in the decisions they make?

## Four stories of value creation

With these themes in mind, I now consider four companies engaged in commercial applications of their own GMEs, focusing on their visions associated with specific product concepts and the value they bring to users and investors. These four case studies are descriptive, and help illustrate different actors’ needs, concerns and perception of value as the development of each company’s software products unfolds. They are built primarily using online publicly visible resources, supported by interviews with the company CEOs (except Endel) and participant observation (in the case of Uncanny Valley).^4^

### Endel—making the case for functional music 

Endel (https://endel.io) is a mature music technology start-up, which provides subscription-based functional music experiences; music specifically targeted at supporting relaxation, focus, sleep and other mental states. It has received over $20M in funding (https://www.crunchbase.com/organization/endel). Endel users can tune into a number of “scenarios” via a phone, watch or desktop app, which can also utilise movement, pulse or other physiological data to modify the music in realtime. These scenarios include artist collaborations with well-known artists such as Grimes and techno veteran Richie Hawtin.

Endel are far from alone in vying for paying consumers of functional music, which has a long history. Playlists on popular streaming platforms dedicated to sleep, focus and relaxation have huge play counts and are thus an important source of revenue. Importantly, here, the identity of the artists responsible for this music is often far less significant to listeners than in other listening contexts. Indeed, it may be considered one of many sources of distraction, from which functional music seeks to offer an escape.

Setting themselves apart from the competing producers of functional music, who aspire to get onto high-ranking playlist on major platforms, Endel have set out to provide a more advanced and complete lifestyle subscription service entirely removed from existing platforms, a platform in itself. They make two critical claims here: the first is that Endel’s music is made with a scientific understanding of how music and sound can shape cognition. Amongst other research references, they point to an arms-length research paper claiming to show quantitatively that Endel’s music outperforms others in supporting focus, as measured by task completion (Haruvi et al. 2021). The second is that their music is adaptive and can be driven in realtime by users’ data such as heart rate and movement. Using this dynamic biofeedback, they claim, offers more advanced performance in musical function (better focus, sleep, etc.).

**Fig. 3:**
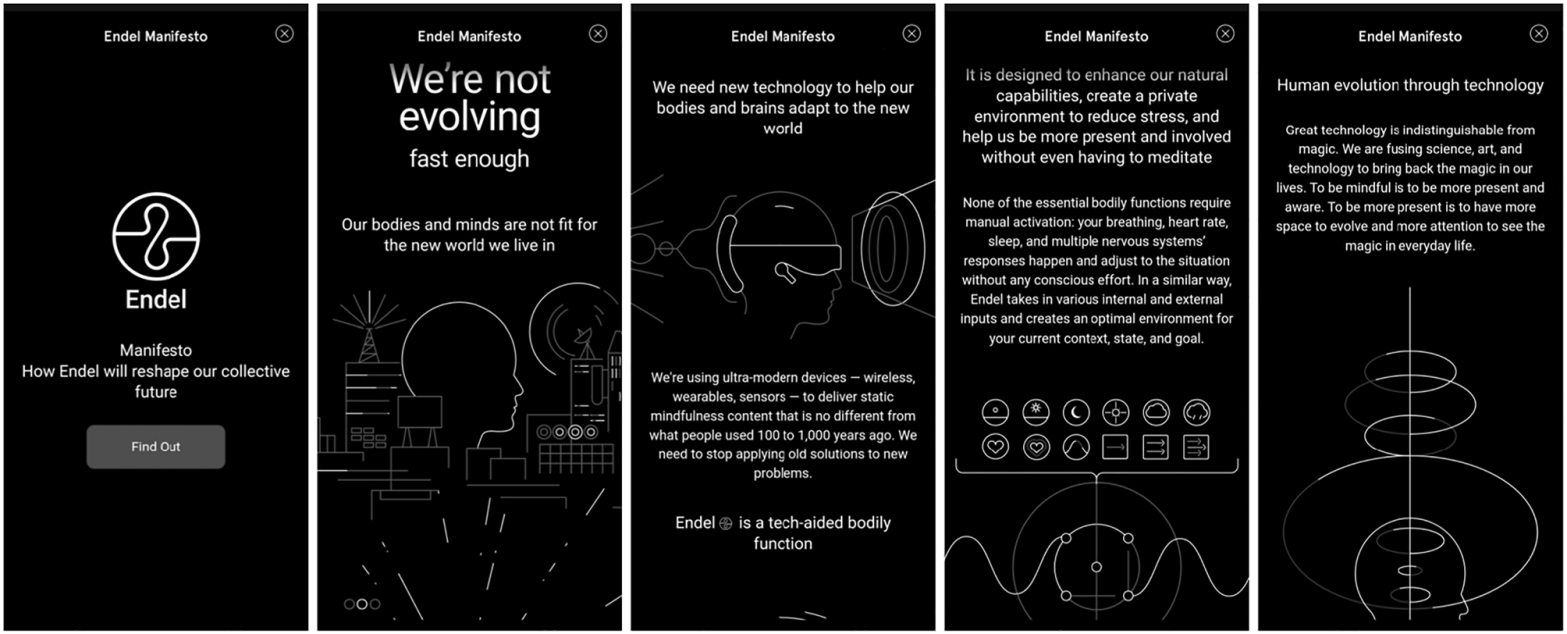
Endel’s manifesto drives a strong cybernetic vision with technology supporting human adaptivity to challenging circumstances. This includes the concept that the user shouldn’t be burdened with thinking at all about

With these offerings, they strongly promote the concept that modern life must urgently be made more bearable and productive using scientifically engineered adaptive music; “our bodies and minds are not fit for the new world we live in”. Their website combines argument-based and impressionistic elements to build this case, presenting an online manifesto (Fig. 3) with sleek graphic design and high production values. The concept of evolution is present throughout, with Endel describing themselves as a “tech-aided bodily function”. The spiel is unequivocal about the enormity of this AI-driven transformation to music experience; it “will reshape our collective future”.

The app design marks an extreme repositioning of the music consumption experience, from the record-store front, where we think of music almost entirely in terms of who made it, to a sensory space completely isolated from issues of authorship and social connection.^5^ The music seamlessly flows through the navigation of the app, giving confidence in the idea that the music is adaptive and ambiently intelligent.

**Fig. 4:**
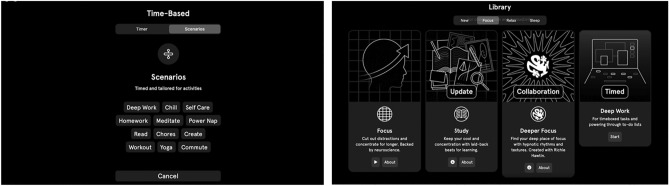
Endel’s app interface is sleek and calming, with no colour, consistent minimalist graphic treatment, and minimal options

Even where Endel has engaged in major artist collaborations, these are nested within the overall design experience, not trumpeted. Within the app, the experience design remains primary. Users are not shopping for their favourite artist, but controlling, using very coarse options, this “tech-aided bodily function”. There is no colour or photography, only Endel-branded vector graphics, which are carefully woven into the app’s flows (Fig. 4).

Endel’s pitch to be the preferred choice for relaxation, focus and sleep music, drawing a regular subscription fee from users who are probably already paying for other platforms with functional music options, depends in part on the case they make for the benefits of adaptive music and the particular quality of their adaptive music technology. But this belies a complex relationship.

Firstly, their argument is ambiguous. Whilst they make a case that functional music can be better if it is bioresponsive, this seems trumped by their own other case, that the key lies in the expert scientific use of frequency distributions, noise, harmonicity and rhythm. The key academic research they cite refers only to this (Haruvi et al. 2021), not the bioresponsive design, and indeed the scientific literature on the focus, relaxation and sleep effects of acoustic design is much more extensive than that on the effects of bioresponsive systems. Couldn’t most if not all of what Endel achieves be achieved via an existing streaming service? Endel seem to have harnessed this ambiguity adeptly: developing a holistic brand identity as expert providers of functional music, the technical details of the dynamic generative system are considered of little concern to users, as long as their personal experience reinforces this messaging. An important aside is that in line with the Google/Facebook business model, and with other health data product lines like FitBit and Nike Sport Kit, getting this fine grained access to users’ physiological data has additional value for the company (this is true despite the company’s intention to use it).

Another dynamic may be playing into this: people seem happy to utilise two different services where a distinction in use is conceptually valuable. For example, users may like the distinction that they message friends on WhatsApp but colleagues on Slack, even if these tools aren’t hugely differentiated in terms of functionality. They become different spaces with different social associations (one may choose to mute Slack but not WhatsApp when on holiday, and vice versa). Likewise, users may find it conceptually cleaner to separate their functional music needs from listening to their favourite bands, Endel can help them “get away” from the cognitive load of Spotify or other streaming services, which push new releases and mixes and offer a bewildering range of options. Again, Endel’s design plays into this. Even if the streams are infinitely generative, this is not pitched as “infinite choice”. There is no shopfront window or library of options, but instead a very short list of scenarios, grouped according to “now”, “focus”, “relax”, “sleep”, and “activity”.

Endel’s CEO Oleg Stavitsky signals this intent:

“Right now, people go into the app and they browse through a catalog of soundscapes and they choose one and decide what they’re going to listen to right now. This is a very old school consumption pattern. I think what we want to get to is a user opening Endel and seeing just one big play button” (Malik 2022).

It is easy to see Endel as an appealing lifestyle choice for consumers with disposable incomes, especially when pitched as having health and productivity benefits. Endel’s own research (Malik 2022) shows that their main users are young professionals needing to focus (many working from home or in open-plan offices), students, and frontline professionals needing to de-stress after a shift. Arguably, Endel is not just drawing listeners’ attention away from other music platforms, but making the case to its users that every part of the day can be enhanced in some way with carefully designed music.

I am listening to music from Endel’s app, in “focus” mode, as I write this. It is playing a perfect 120bpm (my clock happens to be ticking in perfect synchrony in the background). A very soft 4/4 kick and almost imperceptible hi-hat are the only drums, joined by regular chord washes, a gentle fluttering melody and occasional glissandi and other ornamentation. The music is loop based, but not perfectly repetitive. Something is always changing slightly. The chords are not robotic, sometimes spread and pushed ahead of the beat. There is just enough movement in what is happening to not irritate, but no more. Over very long durations things morph. Everything that comes in does so incredibly gradually. The sound palette is rich and lush but with enough grit (e.g., mild distortion) that I don’t find it sickly.

As a music producer with an experimental orientation, I find the music to be excellent, good moody EDM. It convinces me that Endel are adept at creating music that is good to focus to. At other times I have heard what I think are wandering generative melodies, stereotypically shapeless, that convince me there is low-level generation of musical notes in Endel’s system, but leave me wondering how important this is to the overall effect.

I infer that Endel’s GME is largely based on the arranging of pre-prepared audio samples, but with the possibility of adding more adaptive granular generative elements (such as specific note sequences). Since it can adaptively adjust its tempo, and presumably does so running on a user’s device, not as a live stream for each custom user, it needs to carefully manage the computational cost of realtime audio generation. This could be achieved with realtime time-stretching of audio, but that can produce audio artefacts, and Endel seems to place strong emphasis on audio quality.

### Aimi—re-envisioning artists relationship with audiences through infinite streams

Aimi (http://aimi.fm) are a start-up company operating in a similar space to Endel but with a key distinction being that they are much more artist-focused. They have received $20M in Series B funding (https://www.crunchbase.com/organization/aimi-e92a). They also offer never-ending generative music experiences that fall into two categories: those created by named artists, and those created by Aimi, with mood tags such as “serenity” and “flow”. Also, although Aimi’s key branding and messaging appears genre-agnostic, their current offering of artists falls exclusively within techno, house and other related EDM styles.

While Endel have been careful to market their music as a health product without named authorship, except in certain “collaborations” with artists, Aimi have championed a roster of artists. In doing so there is an implicit association between Aimi and a record label or agency, though the artists are simply *using* Aimi’s platform and are in no way “signed” to Aimi. They also draw associations with radio; in their domain name’s use of the “.fm” suffix, and in the creation of experiences that feel like live-streamed DJ sets. However, Aimi do not explicitly draw this association themselves. Furthermore, this is a topic in flux as Aimi develops. In early 2023, it temporarily removed its artist roster, and returned to a simplified selection of streams, but the roster has since returned. Meanwhile, at the time of writing, it is in the process of releasing a creator tool to help electronic musicians produce tracks, and a distribution concept where creators can contribute elements to Aimi’s managed live streams.

**Fig. 5:**
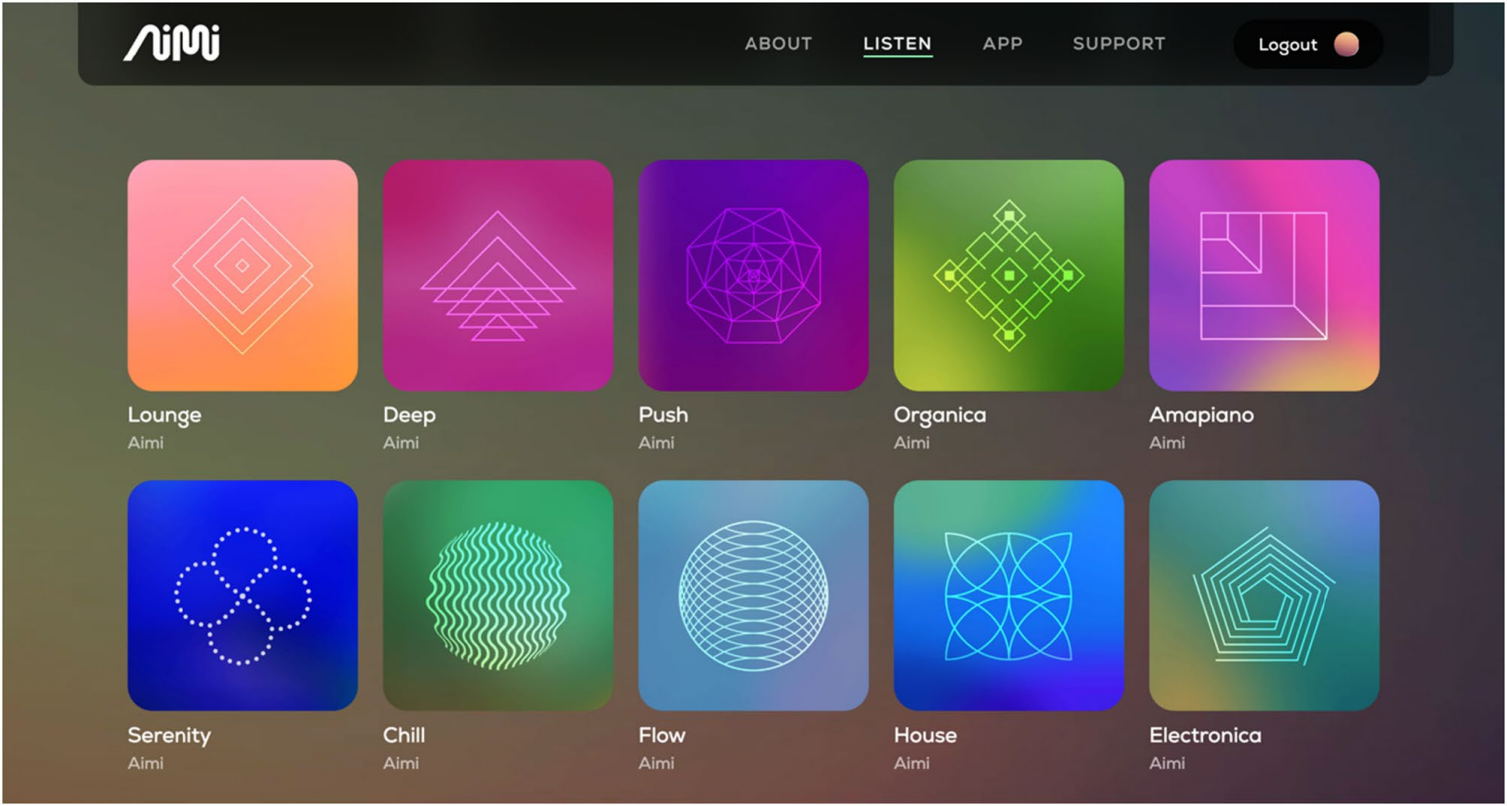
Aimi’s in-house selection of infinite streams, combining some functional identifiers (serenity, chill, flow, push) with genre tags (house, electronica, lounge)

Aimi’s original roster of hand-picked artists were a focused group of techno and house producers. As a judge of their audience numbers, when counted (in Nov 2022), there was one artist with 2 million monthly listeners on Spotify, 15 other artists with over 100,000, another 27 artists with over 10,000, and another 10 artists with fewer listeners. The roster was 86% male, largely solo producers, with a handful of duos (Fig. 5).

Although functional music is a core pillar of their offering, Aimi clearly differentiate themselves from Endel’s values with this artist focus. They strongly push artist and listener-related values that stress frictionless accessibility: they pronounce that the music will always be free and there will never be advertisements. They trumpet the passion artists have for their music and the passion listeners have for the artists. In a podcast interview, Aimi’s CEO and founder Edward Balassanian describes the artists most interested in creation with Aimi as being artists with “art at the heart” (Sparrow 2022). He stresses the slow-build that Aimi has undertaken to engage with artists and their fans, via one-to-one network building within the genres they have targeted. In addition, since some of Aimi’s music production AI is specifically trained on Aimi’s original roster of artists’ production techniques, Balassanian stresses Aimi’s commitment to fairly remunerating artists whose creative skills have been modelled in AI tools. This references an important public debate about fairness an attribution in the use of artist data for training ML systems.

For the listener, they emphasise the ease of selecting music with taglines such as “experience effortless listening”, and “just press play”, providing free and ad-free music.

Aimi’s business model is less clear than Endel’s, given their promise not to charge listeners for the music—exactly Endel’s strategy, and a bold take when the topic of fair remuneration for artists is heated. Their vision for the “future of monetizing music” is relatively traditional for a software company, focusing on their forthcoming paid creator tools. But their generative streaming product, like Endel, also places focus on the power to adapt music in response to data, but with a stronger focus on commercial situations, such as shop sales, where the music could be driven by customer activity.

**Fig. 6:**
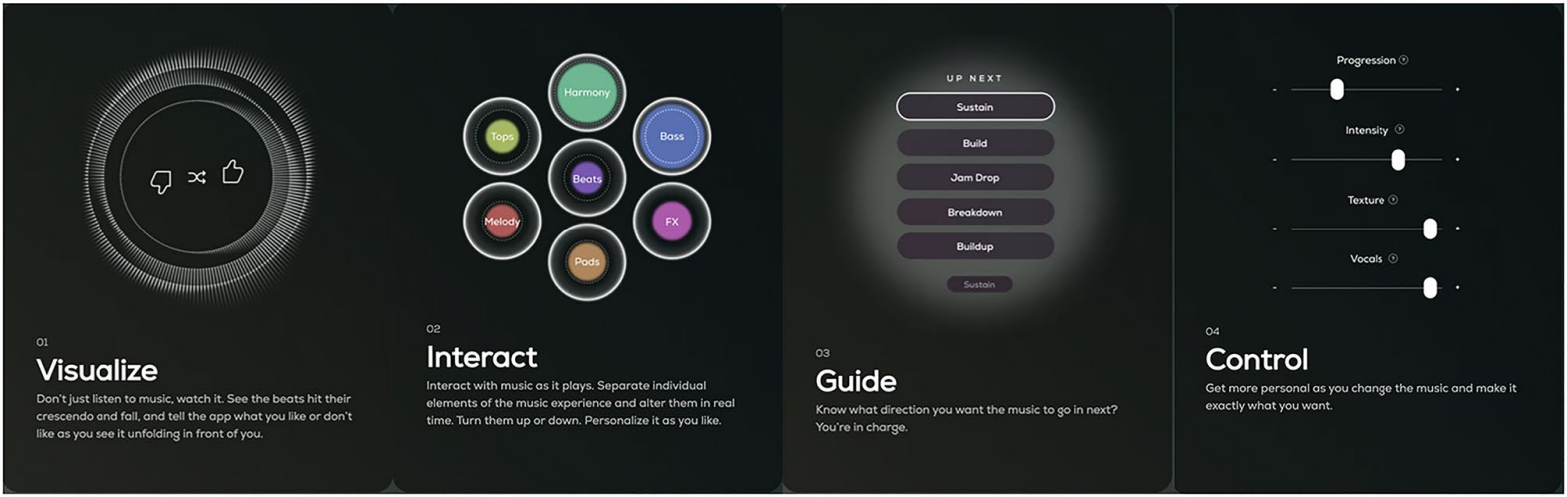
Aimi’s app interface elements, as advertised through their website, indicating some of the possibilities for user control of infinite streams

Further, Aimi’s grander plan involves enriching the artist-audience relationship as an ecosystem formed around their GME. Their player app allows users to interactively control Aimi artist streams, mixing specific elements, and changing abstract parameters such as “intensity” and “progression” (Fig. 6). Balassanian explains that they envision a special type of user emerging, the “curator”, part artist, part fan, who, like a DJ, manipulates artist streams, and becomes part of a production ecosystem. Thus, Aimi’s monetisation plans also involve creating such forms of active engagement with the music and with musical cultures. Critical to the discussion below, it is important to note that none of this is tested yet in good “product market fit”, and to note that there are several potential strands that might get locked into place as this business model develops.

Like Endel, I find the music experience to be impressive and very listenable. The music follows sensible transitions. The mix is coherent. Occasionally, a drop down seems to come out of nowhere or does not build any intensity before it drops back, as you’d expect from a dance music track, but it is never jarring and never sounds wrong. There are fewer flourishes and breaks than in the recorded work of the same artists I listened to, but there are lots of interesting transitions that are not just simple four-bar changes, one-hit embellishments trigger drops, elements fade in and out in interesting ways, and tracks seemingly progress through A-B structures. Above all, the music’s endlessness reminds me of EDM DJ mixes where the relentless constancy of the music can be appealing, putting you in a “zone”, often described as “hypnotic”, but can also become too entrenched in the background. But equally, the relentlessness of the streams becomes striking—they can lack development. The offer of infiniteness begs comparison with other forms of music listening that this experience erases: the radio DJ’s shout outs, their decision to change up the tempo, the natural coming-to-an-end of a show or set, and the sudden appearance of a recognisable hit. How long might someone leave one of these streams running? Could a shop keeper or hairdresser decide to just lock in a certain stream for the long term?

### Splash—empowering amateur music creation

Splash (https://www.splashmusic.com/) (formerly Popgun) is a start-up that uses AI with a mission statement of “bringing the joy of music making to everyone”. With $23M in funding to date (https://www.crunchbase.com/organization/popgun), it has moved through a number of product iterations. At the time of writing its primary product is a game on a popular 3D gaming and social platform, Roblox (https://www.roblox.com/), where players, largely teenage children, can perform music, skate and dance in a virtual music club and skate park environment. Players gain status points from their performance in these activities, and as they gain higher status, gain access to more resources such as audio sample packs. Like many games on the platform, the game combines social and light competitive elements.

Splash’s CEO Stephen Phillips explains how he came up with the game when watching his daughter playing other games on the platform, enjoying how she was immersed and socially engaged, and noticing that there was no existing music-based game on the platform. He envisioned the game as a space where kids can find the confidence to perform, and take pleasure in performing, to play at being famous (where “fame” is “gamified” through points and access to new music resources), but without the obstacles to doing so. The Splash website states: “Splash is the world's best virtual music festival. This never-ending festival brings music and gameplay together in fun new ways. Everyone can feel like a star by using our AI instrument to perform music on stage and interact with live audiences.”

Splash provides tools for players to be able to mix music tracks from a selection of readymade loops. New users entering the game are presented with the chance to perform once they have familiarised themselves with the club environment. They can then choose music “packs” by genre. When performing, users can activate and deactivate different voices and sections on a two-bar cycle, creating music performances. These are rated by other players. They can also be shared outside of the game environment. Based on the game’s Discord discussion server, Splash has a large number of active and highly enthusiastic users who get together in parties in the Splash environment.

**Fig. 7:**
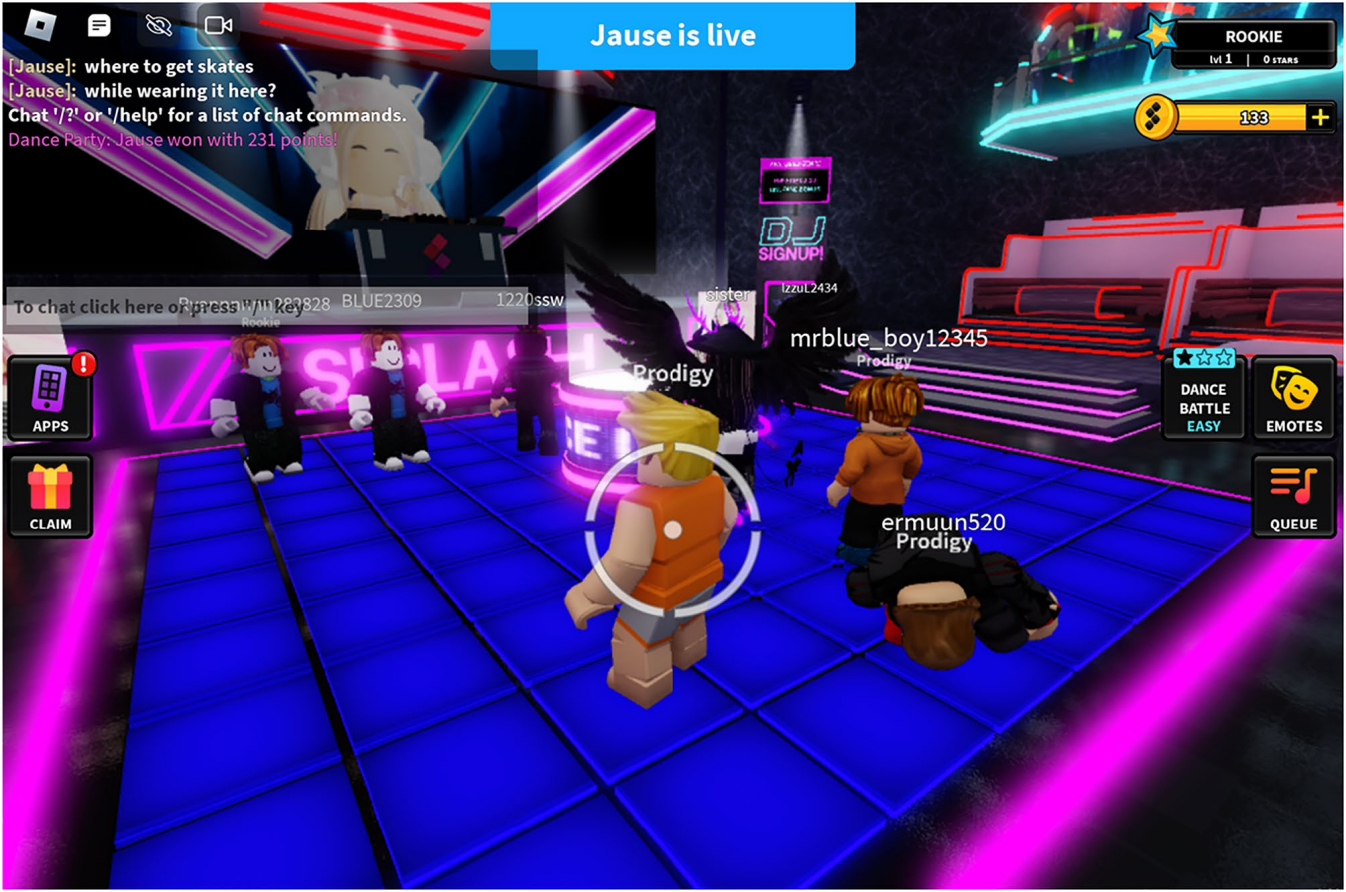
The Splash Roblox game. Users can perform dance, music and skate moves in a virtual club and skate park environment. Success at these performances, rated by other users, increases points and unlocks new music packs

Interestingly, this interface makes no discernible use of advanced machine learning (ML), yet as a company, Splash has done significant prior and ongoing work in AI music tools. There are two points here (both of which I will develop later): as mentioned above, whilst the interface is a simple audio mixer, it establishes a scenario in which non-musicians perform simplified music creation through the selecting, mixing and arranging of readymade elements, in which AI innovations can be identified and developed. This is part of Splash’s longer term strategy. And indeed like Aimi, Splash developers indicate that AI is used more behind the scenes, such as in the generation of new custom user sample packs. Additionally, interviewed developers stressed the design challenge that this use case opens up: finding the challenge sweet spot where non-expert users can easily make music, engaging socially as creators, but not so easily that it is not engaging (Fig. 7).

Secondly, the innovation of the Splash game and Splash’s other products comes from a very strong philosophy of agile development within the company. In interview, Phillips describes a method in which the company is very quick to seed rounds of experimental prototype development, and equally quick to shut them down. Several such phases are documented and Phillips says he has killed about 15 products, with the view that start-ups that do well are the ones that can do rapid experiments and take risks. In prior work, this has included a 6-month development of an experimental AI-powered version of Apple’s Garageband software, and their own music generation plugin toolkit, which embeds symbolic music generation tools into other DAWs.

**Fig. 8:**
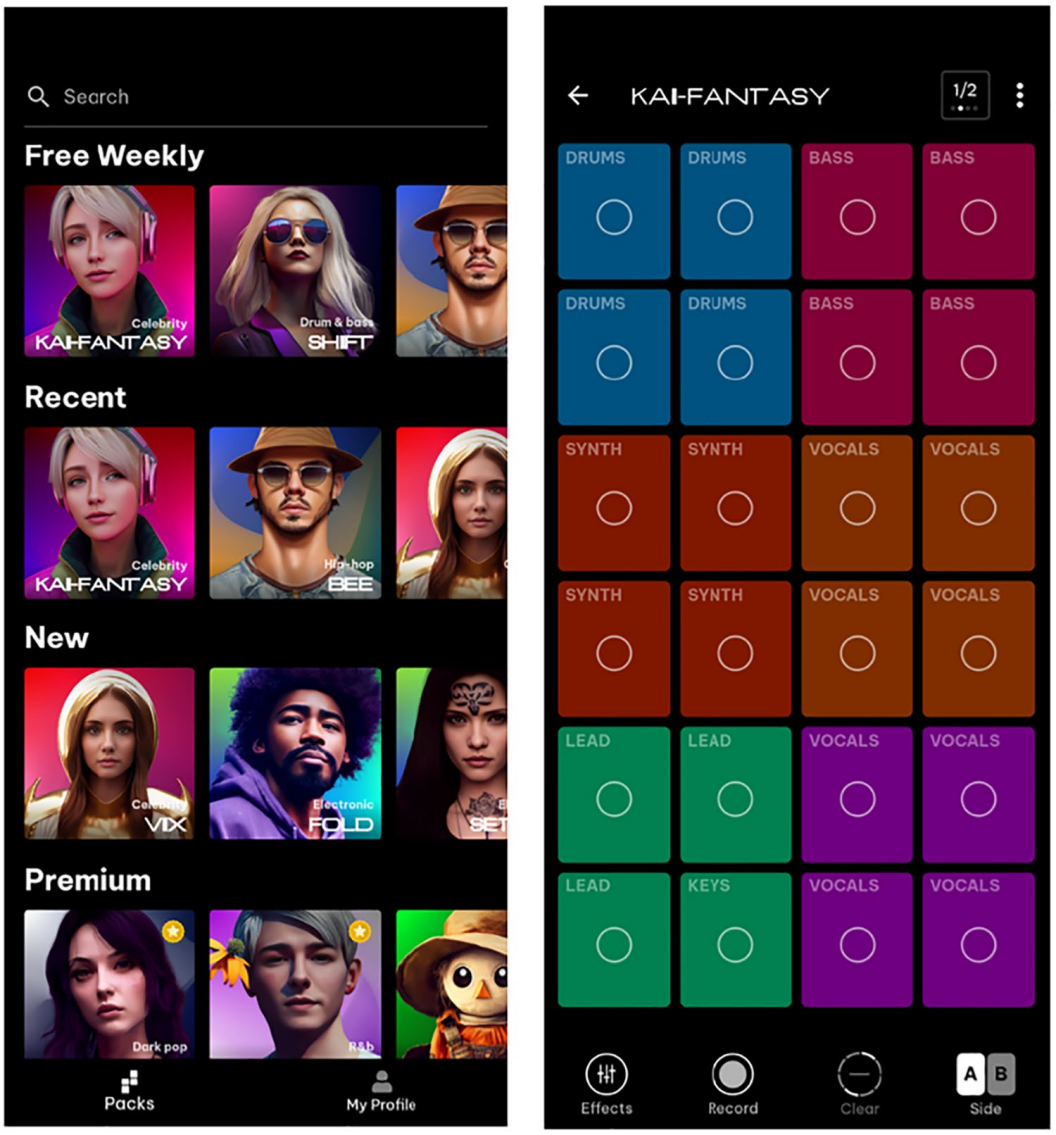
The Splash app. Left, choice of packs. Right, performance interface for a chosen pack

At the time of first writing (early 2023), Splash have announced that the core music creation activity from the game is being moved to an app, with the same basic functionality for music creation from curated packs of readymade loops. Parallel to the Splash game, they maintain a series of virtual AI artists, who appear in the game, forming a wider transmedia storyworld. They have also released demos of AI vocal generation tools, and an AI text-to-music tool that takes a text prompt and creates a hip hop track with original AI-generated lyrics. A new text-to-music tool has since been released (September 2023) (Fig. 8).

As with the other companies, but perhaps more prominent with Splash, given their more extreme and fragmented approach to experimental product sprints, the company’s asset value lies not only in the products but also in its database of music samples, that make up the packs in their game, app and virtual artist products. This was apparent through interviews with the music technology team, who see a core value in their ability to produce music packs for their users. This musical material is produced in-house, at a direct labour cost to investors, and is owned by Splash outright. It is worth noting this labour cost both directs money away from technology innovation, but also enables the company’s other activities. Additional value, importantly, lies with their technical teams, comprising of integrated music and computing/AI skills. For example, in interview, Splash’s CTO revealed his musical rather than software, background and the company’s strong focus on innovation in music/software creation workflows.

### Uncanny Valley—bringing AI music creation into a production music remit

Uncanny Valley are a small production music studio. They are an outlier in this paper, serving a comparative approach, because they are not a start-up and have not received any investment in software development. Their main income is from production music for well-known TV series, for which they have a large resume of major names from reality TV, sports, news and other areas. They often work with experimental technologies and have been actively exploring AI-generated music for over 5 years, culminating in their own GME, MEMU, which they use for bespoke creative projects.

I have had an ongoing working relationship with Uncanny Valley for several years, most recently in three projects that produced a real-time installation artwork utilising MEMU.

Unlike the other companies discussed, although Uncanny Valley explored the commercialisation possibilities of their AI music work, they made the decision in 2021 to keep music production as their core business and to treat their GME and their music AI work as an extension of the work as producers, rather than as a potential software product. This was in recognition of their core values, skill base and strengths as a company. There was too much risk in exploring unknown products, without strong experience in start-ups and commercial software development, and this was a threat to their company’s existing strengths and values, both in terms of time commitment and in terms of impact to their brand.

On this, Uncanny Valley commented that their brand as a production studio often benefited from an association with AI and other cutting-edge technologies. They cite modern car companies as an example, who increasingly want to pitch their cars as advanced entertainment environments. However, they note that for some of their clients AI is not a productive association or goes too easily over people’s heads. Subsequently they choose carefully when to pitch AI-oriented ideas to clients who have not solicited them. They also noted that their physical location has impacted how easy it might be to develop software commercialisation opportunities as opposed to major start-up centres like San Francisco, Berlin and London.

Uncanny Valley have undertaken AI music brand engagement projects with companies including Telstra, a major Australian telecoms company and KPMG, a major accountancy firm and most recently (with myself) in a commission from the Sydney Opera House to “turn the building’s realtime data into music”. These engagements involve producing concept videos showcasing original uses of technology. For example, for Telstra they produced an AI-generated remix of the famous Australian hit *You’re the Voice*. More recently, they licensed a dedicated generative stream, designed as a focus experience similar to Endel, to a major radio and media firm, SCA, whose app was developed to aggregate the firm’s large roster of radio stations with additional podcasts and other audio material. The firm considered Uncanny Valley’s GME an interesting experiment that was worth exploring both as an immediate way to create a dedicated and distinct focus channel on their app, but also as a quick way to dip a toe into the generative music space, with a mind to seeing what might be possible and what parameters arose.^6^

Thus, Uncanny Valley bring a very different perspective for comparison to the other companies. They are engaged in the long-term production of a GME but continue to work as a production company and not as a technology company producing software products. Commissioned projects support the accumulation of capability and prestige in this space, and some projects such as the SCA collaboration have the potential to expand into more significant commercial projects.

The MEMU system is a relatively simple Python program that selects, tunes, cuts up and mixes audio segments according to a simple set of rules and manual tags. It was built primarily by Uncanny Valley co-director Justin Shave, who is also the company’s lead composer. Thus, the system and audio are “in-house” in the extreme: developed largely by the same person.

## Analysis: pairwise comparisons between companies

**Fig. 9:**
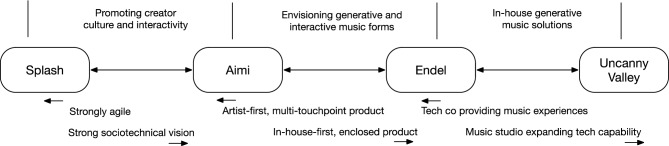
Pairwise comparisons between companies. Comparing Splash and Aimi, both focus on bringing digital interactive musicking to musical cultures. Splash presents as having a much higher appetite for agile development. Comparing Aimi and Endel, Aimi matches much of Endel’s technological vision but with a much more complex ecosystem connecting artists and fans, compared to Endel’s more in-house approach. Endel also has secured a clear product-market-fit with the relatively simple concept of easy and “proven” functional music, and is able to engage in a higher level form of blind search, from the starting point of a developed GME. Comparing Endel and Uncanny Valley, Endel have a more mature GME with significant development resources, while Uncanny Valley represent a production studio bringing generative technologies into their repertoire, but both companies still work largely in-house

We see each of the four companies studied in this paper at very different places in their innovation journeys. It is fruitful now to develop the themes introduced earlier through a set of three specific pairwise comparisons that illustrate the key discussion points developed above (Fig. 9).

### Aimi/Splash—comparing the degree of agile and open-ended search

Aimi and Splash can be considered at a similar stage in their development. Splash visibly pursue an aggressive agile innovation strategy, clearly articulated by the CEO and evidenced by the speed at which they have moved through a series of products. Having done serious development of advanced music AI products, with a dedicated music and machine learning team, their fast-moving approach led them to switch momentarily to a product that had no immediate use of advanced AI, yet still spoke to their narrative of using AI to transform music experience. It also significantly leveraged their growing repository of loop packs, their ongoing development of a GME infrastructure, using a simple loop mixer. Importantly, it provided a space for them to build a relationship with a key user demographic of musically aspirational teenagers, and gain knowledge relating to their vision of how AI can support teenage self-expression and creative social engagement. Splash are in the process of releasing new AI generation tools (based on more recently emerging text-to-audio generation technology) and further developing an app version of their engine. This has taken significant background research in ML and builds on rapid advances in the research sphere.

In contrast, Aimi present a far more targeted vision on a specific product ecosystem, emphasising this consistency through artist and listener values and a singular product offering. In this, they appear more strongly focused on consciously envisioning a transformed musical culture. This is reinforced by their building of a roster of engaged artists, positioning them more ambiguously by infusing their status as technology company with notions from creator communities, record labels and radio stations. Aimi’s release of a new app that enables user interaction and expands their services to artists beyond their curated roster marks a key moment to test experimental designs and gather user data, but the take up of this service is yet to be determined.

In both cases, the company faces immediate challenges in understanding which of a wide range of possible interface designs will best engage their respective user bases. Aimi’s challenge is considerable in having to satisfy both artists and listeners, bringing them on a journey of rethinking established music production, distribution and listening practices. The artists must be satisfied that having an algorithm arrange and mix their music does not significantly undermine the quality of the music: arrangement and mix are far from peripheral creative tasks in EDM, often defining an artist. Aimi’s GME must either do this well automatically or be sufficiently controllable by the artist that they can ensure it sounds the way they want.

Phillips and his developers’ descriptions of Splash’s agile strategy present it not simply as jumping around between different product ideas, but as a cumulative process of developing the company’s knowledge, capability, and profile, for example with technical capabilities, and an understanding of generative music user experience, being recurring concerns across a range of products. The game application is a detailed study of a user experience concept in which, again, music is reconceptualised. However, in this agility, Splash offer significantly less consistency of vision and seem less committed to following through on their ideas, something which may undermine the perception of their values, and reliability.

Likewise, Aimi’s more consistent strategy does not mean there are not significant “blind” phases, and many R&D experiments being undertaken, albeit within a narrower field given their more consistent vision. Aimi’s search is more tightly constrained by a non-varying goal, whereas Splash’s goal is more overtly variable. For both companies, regardless of how open they are to different product outcomes, much of this search may have a common goal of finding “GME efficacy” in terms of how music, technology and specifically AI engineering skills are combined in innovative teams.

### Endel/Aimi—comparing different stages of experimental product development

Endel is the more mature of the four companies. It has successfully built a strong profile as a functional music service, winning over a large cohort of users, presumably achieving product-market-fit. The massive repositioning of music devoid of the rich shopfront of artist selection and engagement, wrapped into a minimal musical experience, successfully supports the brand identity. This is in some ways comparable to the company Muzak who experimented with early forms of functional music including the use of “stimulus progression” to enhance workers’ productivity. Muzak famously became synonymous with blandness in music. Endel have so far been successful in avoiding such associations through a combination of an enchanting cybernetic narrative and, arguably, the quality of the music. In doing so they have tapped a key decision point in users’ lives: when they need to focus, relax, sleep, etc. do they reach for the chaotic shopfront of Spotify or Apple Music, with the high cognitive load of choice, or do they reach for the simplicity of Endel? The functional utility of AI here is ambiguous, but its role in the narrative is key: the music adapts to you, so you can focus on the thing you’re doing (in some ways this is a logical extension of the concept of music recommendation).

Endel’s engagement in blind search is therefore less visible. What is key here though is what comes next, now that Endel’s engine has reached a degree of maturity. Endel describes a series of integrations (https://endel.io/integrations) with cars, consumer electronics, health and wellness, retail spaces, “mass meditations”, and work and education, that extend beyond the regular personal app experience. Here, we see Endel embedded in a more advance phase of search, that builds upon a secured initial viable product. In contrast to Endel’s very focused core product, these various integration concepts are presented as an unordered list of possible music reimaginings. Few offer very much detail, some may be very simple concept experiments that help furnish this vision of a great diversity of application potentials. Any one of these may become the real killer application driving Endel’s success, making them the 100 × company and new market owner their investment logic demands.

Aimi is comparable, though at an earlier stage of development. They envision a myriad of novel applications that can be serviced by their basic recipe of GME, artist-created content, and interaction scenarios. Developing evidence of their products’ extended application potential in new scenarios is a key goal. But with a far more complex end-to-end ecosystem of music production and experience, this goal must also compete with other necessary proofs-of-concept, each involving additional development commitment, to arrive at a clear picture of that ecosystem.

The major difference is that Aimi’s forthcoming app provides the user with a vast amount of creative control over the music which is an untested concept in two ways: firstly, in how users might respond to such choice, and secondly in how artists might accept or reject the notion of their work being creatively manipulated, with no authoritative original form to refer to as the master. Unlike Endel, then, Aimi’s business model is significantly higher risk in terms of an understanding of the user experience and the work involved in building it, though the payoff may be greater in the end.

### Endel/Uncanny Valley—comparing different approaches to integrated in-house music-and-software development

Lastly, comparing Endel and Uncanny Valley is informative. Both have in common that their GME and production of musical assets are done in-house (i.e., not involving 3rd party artists^7^), meaning they share the task of developing an integrated process of infinite music production, with in-house producers developing materials that are well coordinated with the GME. Both subsume the artist, Endel as part of a carefully constructed product offering, Uncanny Valley more simply as an extension of their work as a production music house. The in-house integration reduces some of the issues faced by Aimi, who must find ways of fitting in with 3rd party artists’ expectations and workflows. Both Aimi and Splash are also seeking interface designs to allow end-users to play with music streams. For Endel and Uncanny Valley, the end-user is simply a listener.

The differences are of scale and orientation: an ascendent platform, Endel, versus a small two-person team, Uncanny Valley. Yet, both are in the open-ended search for new applications for their engines through a variety of spin-off experiments. For Endel, this is an active, broad search through the world of possible integrations with major partners. For Uncanny Valley, it is highly constrained by the individual commissions that come in from clients with specific interests in generative streams, combined with their own ability to cross-subsidise experimentation from their core business. At these two levels of scale, though, we see two different innovation and software development processes. For Endel, the GME is managed by a large team, carefully versioned and documented, with the potential to generalise a powerful engine. For Uncanny Valley, there are between 1 and 3 developers working for short periods of time when specific projects allow it. The resulting engine is more bespoke and unlikely to scale up to a more general framework (though not impossible as the code base accumulates).

## Discussion

This comparison of four companies struggling for success in GME-based products, with their differences and commonalities, provides a portrait of the emerging landscape of GME innovation. The three start-ups share ambitious visions for how their work could transform wider music culture through the realisation of specific new forms of generative music production and consumption. They are marked by differences in how those visions are managed with respect to the risk and uncertainty inherent in their endeavours.

This paper considered these case studies with two questions in mind: Q1 how do start-ups manage innovation in such a speculative field? Q2 how does their own conception of value play out in the decisions they make? I consider Q1 first.

Endel, the most mature company, has established a clear and simple value proposition as the go-to service for functional music, manifest as a focused subscription-based platform. This proposition is communicated through a strong cybernetic narrative fusing AI and evolutionary themes, but without the high risk of depending heavily on AI innovation or radically new forms of community building to achieve their goal. Arguably, AI is a peripheral contributor to their product’s value in practice. Thus, with respect to innovation, Endel seemed to be initially conservative in the risk they took with exploring AI product concepts. The primary value of Endel’s product is arguably the quality of its music, not its AI. By making music in-house, they have ensured this quality, and have also avoided having to build creator tools or listener interfaces with complex end-user design requirements. But their resulting mature framework and existing user-base makes them well-placed to open up more speculative experimentation in adaptive music integrations, and this is underway through ambitious experiments. They have adeptly manoeuvred to the point where they have brought a mature GME very early to market with established product-market-fit and the freedom to speculatively explore its wider application value.

Aimi and Splash are younger companies, both well-funded, and both offering impassioned visions for how their work will improve music culture through technology. Yet their founders present quite marked differences in reconciling their vision with different levels of agile practice. Aimi presents the more committed and fleshed out vision of an enhanced music culture in which they connect electronic music artists with audiences in new technologically-mediated experiences. But this vision comes with a complex entangled space of technological, sociocultural and design challenges that the company must navigate. Splash have a more ambiguous vision and overtly express a culture of rapid, agile development, moving between more modular product ideas in rapid sprints, a more aggressive search for a minimal viable product and product-market fit. Despite these differences, both companies are constantly accumulating forms of value, from databases of audio assets to new approaches to integrating software development, AI, and music expertise in their teams. They are empowered by generous funding to do so, but with the expectation of converting these accumulated assets into profits.

Uncanny Valley, the outlier, abandoned their product development ambitions, choosing instead to stick with bespoke GME development for specific creative commissions in line with their core business as a production music studio. Their GME is funded to tens of thousands of dollars rather than millions, but still draws on a wide ecosystem of available AI music software and illustrates how diverse the GME innovation space is. But they represent how GME development exists in diverse forms besides technology firms, and their work is typical of production studios increasingly incorporating creative technologies development in a studio practice.

This shows how each start-up product vision remains in tension with their product reality in various ways. Endel overstate their AI narrative as part of a rich imagery that has seemingly played well with audience engagement. Aimi face a complex challenge of better understanding the emerging user culture that will serve their designs and achieve the vision of a new ecosystem in which engaged “curators” perform a new role. Splash manage an ongoing disconnect between creating experiences that gamify musical success and the reality of supporting musical expression. Like Endel, their success seems more decoupled from their AI investment than their vision may make apparent, but this could pivot at any time as they introduce diverse products.

The second question (Q2) concerns how companies develop and perceive their own value over time, not only in their immediate product vision or software offering but in all its forms, from assets to culture. These case studies have attempted to capture ways in which, on their journey to a product vision, the *residual* value that gets built—algorithms, datasets, teams, userbases—is an immediate but hidden factor that drives future development, offering new opportunities for success that may, in doing so, ultimately present opportunities that are contradictory to their own vision. Thus, Endel can be seen as successfully prioritising the creation of a user-base of functional music listeners, in order to facilitate a more in-depth generative music product search. Splash’s strongly agile approach to products can appear eccentric but belies a more consistent and steady accumulation of developer and AI capability, whilst nurturing a user-base of young gamers, and the cultural capital of their AI artists. Like Endel, the users may be there for slightly different reasons than the quality of any AI music product—in Splash’s case, the environment is above all a “festival”, a fun social space. Aimi can also be seen as embodying some of these elements but playing a longer game: developing GME capability (in both teams and software) and nurturing a user-base of electronic musicians, who also provide rich user-experience data about musicians’ attitudes to GME use.

This portrait of four innovation journeys is characterised by competing agencies, portraying a situation of dynamic sociotechnical emergence. CEOs and investors have extraordinary power to influence musical culture, employing vast capital resources with which they can enlist large bodies of users into their technology ecosystems, using product visions and narratives of the future of music as their primary means to enact that power. Thus, on one hand, it is essential to critically examine how that power is managed, who has access to it, and what drives it in commercially led sectors. But that power is also balanced by two forces. The first is the unfolding product reality: does the technology work, is the user experience design successful, and so on. Here, products are collectively shaped through the emergent phenomena of iterative human-centered design and commercial competition. The second is the external commercial sphere, where individual visions are simply usurped under acquisitions or other transitions. Here, product visions may disappear altogether but the technologies they produce remain, exapted to new application areas that may not share the socially positive principles found in the original vision.

With these considerations in mind, a comparison between such product visions and Hodgson’s notion of “imagined metrics” (Hodgson 2020) is fruitful. Hodgson describes how music technology start-ups frequently deal in over-exaggerated figures for potential revenues, with investors, CEOs and employees sharing in the imagination. The product visions considered in this paper are not so much exaggerations as simply inherently speculative constructions of possible musical futures. To investors, they inspire revolutionary potential. But in the interests of agility, they must remain open-ended and ambiguous, iterated within companies as well as through user-centered development. In so far as pivots and acquisitions are concerned, product visions are ultimately separable from the real assets of the company, with all parties aware of this potential eventuality.

What, then, do these case studies point to as potential impacts on music culture? Endel current maturity in product-market fit is notable here in that to some extent its bid, seemingly successful, is not simply to compete with other platforms, but to fill more of our day with music, potentially growing the entire music market. But it also has the potential to replace long standing cultural practices in which music listening involves audiences connecting with artists, related to forms of cultural cohesion, with a form of industrialised music, making us feel relaxed, and potentially disconnecting us from music’s social value in its hyper-personalisation. Even if the music is a significant cut above faceless ‘elevator’ music, it is still the production of a corporation. Were a significant shift towards use of such platforms to occur this could simultaneously disempower artists and devalue music’s power to connect.

In its reimagining of musicking as a “democratised” process, with a significantly reduced barrier to entry into an individualist–competitivist sphere, Splash has similar potential, but pulling in another direction: this time predominantly social but potentially lacking deep engagement insofar as creative fulfilment is concerned. It seemingly has the potential to nurture creative music practices in which there is a high degree of dependence on the platform, and in the Splash environment, the company potentially controls the means to make music, the music artefacts and the variables that influence success (as well as virtual artists entirely owned by Splash). But this time the environment is highly participatory, and its lively Discord servers demonstrate users’ engagement. There is good reason to be wary of this capturing of creative expressive performance in an environment that offers seductively easy tools, but there seems to be no lack of cultural vibrancy in the resulting world. This points to an important topic for future research into AI music cultures: distinguishing and comparing value propositions such as deep engagement in music practices with equally abstract and intrinsic value concepts such as social engagement and cultural expression in these new forms of musicking. Here, Splash sit amongst a growing field of companies, most notably Bandlab, who are nurturing Garageband-like music tools offering massive readymade databases of music and very low barriers to the construction of complete tracks. Whilst AI’s use may be relatively constrained at present in these creative technology ecosystems, these tools are AI-oriented in their future thinking, creating myriad pathways to introduce AI into their many creation workflows.

Meanwhile, Aimi, the most artist-focused of the companies looked at (as well as Endel, with their artist collaborations), present a scenario where those artists who produce with the service face potential creative challenges in the control they have over musical output, leaving decisions to be automated by a realtime GME. If Aimi (as with any GME company) were to become a major platform, the minutiae of its GME design would strongly influence how the music we hear sounds. This is not usual in the historical relation between music technologies and music practices: examples include forms of within-platform competition such as the “radio wars”, where producers pushed mixes to achieve increasingly competitive loudness levels for radio. But GMEs represent a radical evolution of music distribution beyond fixed-media recordings. One could consider the rise of GME-mediated music being more akin to the history of website design, where web APIs and common standards dictated the kinds of websites people could make.

More broadly, then, in a world of multiple commercial competitors creating bespoke generative tools, there is, on one hand, a risk of fragmentation in which different GMEs are incompatible and nurture different music cultures, and on the other, the risk of an effective monopoly. If Aimi’s vision plays out, artists would submit musical segments to a GME, and possibly then engage in more or less depth with the scripting of generative behaviours. The web open standards developed by communities may be a good model for alleviating these risks and making a more pluralistic environment, in line with many other calls to avoid market centralisation in AI broadly (Crawford 2021), and creative AI specifically (Drott 2018; Born 2022a, b). This may emerge naturally under market pressures, but may also need to be driven by interested research communities or campaigners.

Furthermore, Aimi, Endel and to a lesser extent Uncanny Valley’s vision have in common that they invite new forms of listening where the taste-making power of recommendation algorithms could conceivably be employed to work at increasingly granular levels, algorithms selecting which components come next or are mixed together. With intense scrutiny on the effect of recommender systems on individual and collective taste-making (Born 2022a, b) such developments will be important to monitor.

In these comments, I have not made connections with the specific minutiae of GME design features, but there is future potential to do so. Beyond the sequencing together of blocks of audio lie significantly more complex musical objects, potentially mediated by AI; segues, “drops”, fills, bridges, modulations, crescendos and so on. Each of the GMEs’ developer teams is tasked with enabling the creation of flexible and powerful music, which depends on such objects. It remains an ongoing issue how effectively any such devices are implemented, and this will be an ongoing concern in the competition for a successful GME as well as a focus for future research in this area.

## Conclusion

In this paper, I considered as case studies three start-up companies and one music production studio, each vying to build GME-based music experiences. I analysed their product visions, seeking to understand how they innovate and how they apply their own conceptions of value. The resulting portrait of GME innovation helps us consider how creative agency is enacted in music innovation in the context of start-up culture, which I view as an emergent product of product visions interacting with technological and cultural realities, neither determined by a pure market nor by a founder’s vision, but in a complex of interactions. I consider how residual value is a strong factor influencing how start-ups realise their product visions, and look at some of the possible ways that the success of any one of these GME innovators might have wider reaching impacts on music culture.

## Footnotes

1 https://sfist.com/2019/06/14/bay-area-billionaire-vinod-khosla/

2 Indeed, the utility of the term artificial intelligence to describe current advanced machine learning systems is in question. https://medium.com/center-on-privacy-technology/artifice-and-intelligence%C2%B9-f00da128d3cd

3 Which seems almost unavoidable; I have already used the word path.

4 Data can be made available on reasonable request.

5 Though the history of streaming platforms includes those who opted for a minimalist, radio-like, experience: “you press play, we’ll do the rest”.

6 From an interview with SCA’s Innovation Manager.

7 Although Endel does engage in “collaborations” with artists, these are few, and appear as major investments where Endel’s in-house team would presumably work closely in the integration into the system. This is different to Aimi’s goal of making a system that integrates artists’ work with minimal human input.
